# Altered metabolism in cancer

**DOI:** 10.1186/1741-7007-8-88

**Published:** 2010-06-25

**Authors:** Jason W Locasale, Lewis C Cantley

**Affiliations:** 1 Department of Systems Biology, Harvard Medical School, Boston, MA 02215, USA; 2 Beth Israel Deaconess Medical Center, Department of Medicine, Division of Signal Transduction, Boston, MA 02215, USA

## Abstract

Cancer cells have different metabolic requirements from their normal counterparts. Understanding the consequences of this differential metabolism requires a detailed understanding of glucose metabolism and its relation to energy production in cancer cells. A recent study in *BMC Systems Biology *by Vasquez *et al*. developed a mathematical model to assess some features of this altered metabolism. Here, we take a broader look at the regulation of energy metabolism in cancer cells, considering their anabolic as well as catabolic needs.

See research article: http://www.biomedcentral.com/1752-0509/4/58/

## Commentary

**Figure 1:**
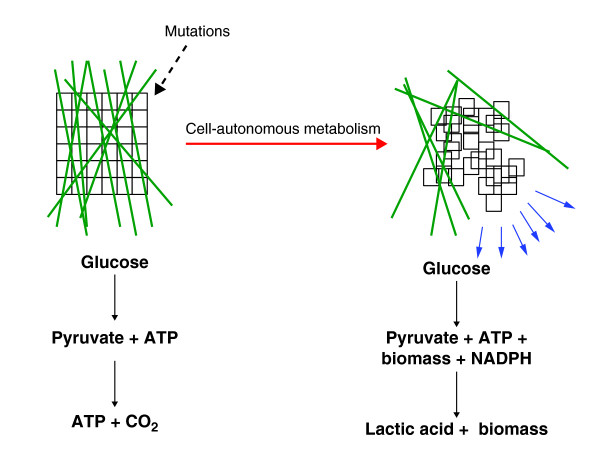
**Cell-autonomous control of growth and metabolism are acquired upon cell transformation by mutation**. Cells in a normal tissue (left-hand diagram) are constrained in their growth by their environment (depicted as green lines). During the development of a tumor (right-hand diagram), cells acquire mutations that allow growth outside the context of normal tissue development. As a result, metabolic pathways are reorganized and metabolism is altered to allow for cell-autonomous growth.

Cancer is a disease of uncontrolled cell growth in which cells acquire genetic alterations that allow them to proliferate outside the context of normal tissue development. In the evolution of this transformation, cells acquire mutations that confer selective advantages for the growth of the tumor. Genetic alterations in many of the known oncogenes are selected to adapt cellular metabolism to meet the requirements of rapid cell proliferation as well as autonomous growth and survival in an environment absent of contact with extracellular matrix (Figure 1). Accumulating evidence indicates that almost every known oncogene regulates downstream targets that are directly connected to metabolic regulation [1]. A detailed biochemical and systems-level understanding of precisely how oncogenes rewire metabolism is essential to understand tumor biology, but concomitantly requires an assessment of the metabolic adaptations required to support the proliferation of cancer cells. Understanding the consequences of this differential metabolism requires a thorough analysis of glucose metabolism and its relation to energy production in cancer cells.

In a majority of tumor types, an enhanced rate of glucose uptake is observed and serves as a reasonable starting point for understanding differential metabolism in tumors. Otto Warburg's initial observation that tumors often metabolize relatively large quantities of glucose predominantly through a fermentative-like metabolism, resulting in lactate production in aerobic conditions (termed aerobic glycolysis), provided the phenomenological foundation for studying altered metabolism in cancer [2]. Rapid progress is being made towards a molecular understanding of why lactate production from glucose gives cancer cells a growth advantage. Paradoxically, cells that achieve high rates of aerobic glycolysis often show relatively small changes in the rate of oxygen consumption in response to changes in glucose uptake; that is, oxidative catabolic flux through the Krebs cycle leading to mitochondrial ATP generation is somewhat independent of glucose metabolism [3].

## Alternative metabolic fluxes support the Krebs cycle and mitochondrial ATP production

Catabolic pathways involving the oxidation of material other than glucose in the Krebs cycle are also involved in cancer cell metabolism. For example, glutamine flux into the Krebs cycle has been directly observed in cancer cell lines and appears to be in part regulated by expression of *MYC *and *TP53 *(p53) - two of the most common cancer-associated genes [4-6]. Additional amino acids such as arginine and glycine, and metabolic intermediates such as fatty acids, can also be metabolized by mitochondrial pathways in certain contexts. These metabolites have transporters to deliver them into cells and are present in sufficiently high plasma concentrations to support their use in catabolic metabolism in mitochondria [7]. In addition, many of the anabolic products that stem from intermediates in glycolysis can ultimately flow into the Krebs cycle, resulting in a bypass of the generation of pyruvate - the end product of glycolysis. This type of glucose metabolism avoids metabolic activity involving pyruvate kinase and pyruvate dehydrogenase, which are typically inhibited in cancer cells [8]. These alternative pathways present many opportunities for additional stages of regulation in the decision to commit carbon flux to anabolic versus catabolic metabolism, and more research is required to understand the origins and tumor specificities of Krebs cycle flux.

## ATP requirements in tumor cells

Aerobic glycolysis is considered a relatively inefficient way of producing ATP, as the alternative catabolic fate of glucose via oxidation in the Krebs cycle and donation of electrons into the electron-transport chain can generate 15 to 20 times as much ATP per unit of glucose. A recent study by Vazquez *et al*. [9] used a reduced flux-balance model to suggest that synthesizing ATP from glucose through aerobic glycolysis is the optimal ATP-generating strategy when a cell is limited by its capacity to maintain enough mitochondrial mass to support sufficient flux through the electron-transport chain. Whether fermentation is an optimal ATP-generating strategy is unclear; however, there are several lines of evidence that suggest that tumor cell proliferation is not limited by ATP availability.

**Figure 2:**
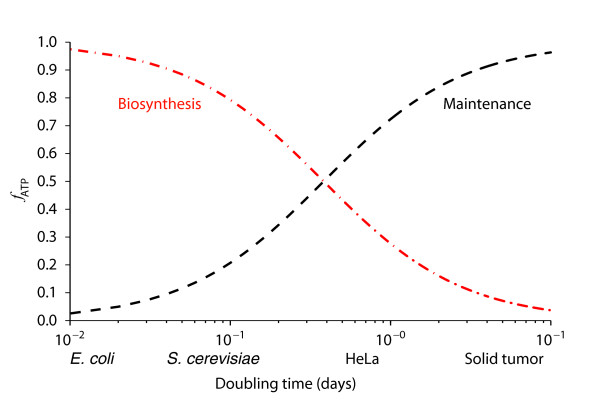
**ATP requirements in cell maintenance and division**. Using a model based on differential equations for growth rates [10], the fraction of ATP, *f*_ATP_, consumed for biosynthetic (red dashed line) versus maintenance (black dashed line) purposes in cells is plotted as a function of doubling time in days. Typical doubling times for unicellular organisms such as *Escherichia coli *and *Saccharomyces cerevisiae *are shown, as well as for typical cancer cell lines (HeLa) and solid tumors.

For mammalian cells, calculations suggest that most of the ATP generated is consumed in basal cellular processes such as the maintenance of concentration gradients through ion pumps and active transport using molecular motors. A simple calculation shows that biosyntheses are not a major source of ATP consumption in tumor cells [10]. As shown in Figure 2, ATP requirements for maintenance and proliferation can be plotted as a function of cell doubling time. For cells that divide on the order of minutes, most of the ATP is used for cell growth. However, for cells that divide on the order of days, such as those in tumor tissue, almost all the ATP is used for cell maintenance. One possible source of confusion about ATP generation in tumor cells is that the apparent lack of demand for ATP in cell proliferation is in contrast to bacteria and other unicellular organisms. These microorganisms undergo rapid cell division on a time scale of minutes and it is estimated that most of their ATP is used for biosynthesis. In all, the calculations suggest that the Warburg effect may not be related to a cell's optimal ability to generate ATP. Furthermore, as we discuss next, ATP hydrolysis can be a limiting factor required to support high rates of glycolysis.

## Stoichiometric consequences of high rates of glycolysis

The stoichiometry of glycolysis imposes chemical constraints when high fluxes of glucose occur. Consider the overall chemical equation for conversion of glucose to pyruvate via glycolysis:

From the equation, it is clear that glycolysis is not possible without sufficient regeneration of ADP and NAD^+^. The reduction of pyruvate to lactate by lactate dehydrogenase is the most ubiquitous mechanism known for converting NADH back to NAD^+^, and this activity balances the very high rate of glycolysis observed in most cancer cells. Efraim Racker noted the problem of stoichiometric ADP availability and postulated that large fluxes through yet to be characterized ATP-coupled hydrolysis reactions were required to balance the cellular glucose uptake rates found in tumors. Futile cycles, more conveniently directly coupled to glycolysis, that hydrolyze ATP were believed to be required to balance high glycolytic flux [11]. One now established example of a futile cycle in glycolysis involves a shunting step in which fructose 6-phosphate (F6P) is phosphorylated to form fructose 2,6-bisphosphate, which is then dephosphorylated back to F6P, resulting in net ATP hydrolysis. Attention has recently been paid to the cancer specificity of these reactions and some studies suggest that the enzyme activity responsible may be differentially regulated in some cancers [12]. Other ATP-consuming futile cycles in central carbon metabolism may yet be discovered.

## Growth advantages of aerobic glycolysis

Although aerobic glycolysis may, by virtue of stoichiometry, be necessary to support high rates of glycolysis, the advantages of this process for tumor cells are complicated to understand. Cell-autonomous effects of lactate secretion are likely to confer advantages on tumors. Lactate may enhance the invasiveness of tumor cells by disrupting normal tissue architecture as well as promoting an environment with reduced pH to evade tumor-attacking immune cells.

A complementary teleology suggests that maintaining high rates of glycolysis is required to reconfigure metabolic pathway fluxes to achieve more efficient anabolic metabolism and cell-autonomous growth. One speculation is that a movement of pathway fluxes towards anabolic metabolism can arise from the effects of differential ATP hydrolysis and redox balance that originate from the demands of the stoichiometry shown in the equation above. These differential pathway fluxes are likely to be dependent on tumor type since different oncogenes are used to regulate different anabolic fluxes.

Cancer cells are limited in their growth by the availability of carbon skeletons needed to produce new proteins, nucleotides and lipids [13]. Furthermore, the reducing equivalents in the form of NADPH required for reductive biosynthesis derive from pathways that are orthogonal to ATP-generating pathways. Multiple solutions to the problem of obtaining sufficient carbon material and reducing equivalents are obtainable, and probably depend on the genetic context of the tumor and its microenvironment. A comprehensive understanding of how NADPH is generated and used in cells, and the predominant anabolic carbon fluxes stemming from glucose uptake, will help to parse the molecular consequences of aerobic glycolysis.

Because of the complexity of cancer metabolism, a better understanding will ultimately require the use of mathematical models. This improved understanding will then allow intervention in the metabolic pathways responsible for tumor cell metabolism. Exploiting these tumor-specific properties presents opportunities for therapeutic intervention in tumor development. The pharmacological targeting of enzymes that regulate this restructured metabolism has recently shown some promise in preclinical studies [14].
